# Assay-aware flow-cytometric interpretation of relapsed B-cell precursor acute lymphoblastic leukemia after sequential CD19- and CD22-directed therapy: A case report

**DOI:** 10.1097/MD.0000000000051427

**Published:** 2026-11-06

**Authors:** Fangxiong Zheng, Juan Dong, Xiaocui Cao, Shuangzhe Li, Tengfei Ma, Jiayi Li, Jinxi Wang, Yuchen Zhang, Zhongxin Lu

**Affiliations:** a Department of Medical Laboratory, The Central Hospital of Wuhan, Tongji Medical College, Huazhong University of Science and Technology, Wuhan, Hubei, China; b School of Laboratory Medicine, Hubei University of Chinese Medicine, Wuhan, Hubei, China; c Wuhan Blood Center, Wuhan, Hubei, China.

**Keywords:** B-cell precursor acute lymphoblastic leukemia, blinatumomab, case report, flow cytometry, inotuzumab ozogamicin

## Abstract

**Rationale:**

Sequential CD19- and CD22-directed therapies can make relapse immunophenotyping assay-dependent. Surface and cytoplasmic antigen results address distinct clinical questions and should not be interpreted interchangeably. This case uniquely integrates sequential target exposure, discordant surface and cytoplasmic findings, exclusion of mixed-phenotype acute leukemia, and interpretation of a post-inotuzumab ozogamicin (INO) population between the assay limits of detection and quantification.

**Patient concerns:**

A 73-year-old woman with B-cell precursor acute lymphoblastic leukemia received a 14-day course of blinatumomab and later developed marrow relapse after a prolonged gap in specialist follow-up. The available records did not permit reconstruction of the complete treatment-selection history.

**Diagnoses:**

At relapse, the blasts were positive for cytoplasmic CD19 and CD79a, lacked surface CD19, expressed weak surface CD22 and CD13/CD33, and were negative for cytoplasmic MPO, CD14, and CD64. These findings supported relapsed B-cell precursor acute lymphoblastic leukemia with aberrant myeloid-associated antigen expression rather than mixed-phenotype acute leukemia.

**Interventions:**

The records documented blinatumomab exposure followed by INO-based salvage on three occasions. The reason for the abbreviated blinatumomab course and body-surface-area-based INO dosing could not be reconstructed from the reviewed records.

**Outcomes:**

Posttreatment marrow contained 0.5% blasts and a low-level abnormal population of 0.007% (approximately 30 events), which was above the assay limit of detection but below the limit of quantification. Surface CD22 was not detected on this low-event population with the S-HCL-1 assay. At later assessment, no previously identified leukemia-associated abnormal population was detected under the reported assay conditions.

**Lessons:**

After sequential antigen-directed therapy, lineage assignment and therapeutic-target assessment require repeated, assay-aware interpretation. Cytoplasmic antigen positivity may support lineage assignment but should not be equated with surface therapeutic-target availability. A low-event multiparameter flow cytometry population below the limit of quantification should be described qualitatively. A single posttreatment sample cannot establish lineage switch, antigen loss, treatment causality, or durable disease control.

## 1. Introduction

Blinatumomab and inotuzumab ozogamicin (INO) have expanded treatment options for relapsed or refractory B-cell precursor acute lymphoblastic leukemia (BCP-ALL), including in older adults. Their use also makes the antigenic context of relapse clinically important.^[1–4]^

After CD19-directed therapy, relapse may show absent or reduced surface CD19, discordance between surface and cytoplasmic antigen detection, or other phenotypic changes.^[5,6]^ We describe a relapsed BCP-ALL case in which distinguishing the disease from mixed-phenotype acute leukemia (MPAL) and interpreting a very-low-level post-INO population required explicit attention to assay conditions and analytical limits.

Prior reports have primarily described CD19-negative relapse after blinatumomab, outcomes after sequential blinatumomab and INO, or reduced or lost CD22 expression after INO.^[5,7–9]^ In contrast, this case brings together sequential CD19- and CD22-directed therapy, discordant surface and cytoplasmic antigen findings, exclusion of MPAL, and interpretation of a post-INO population falling between the assay limit of detection (LOD) and limit of quantification (LOQ). Its educational value lies in demonstrating that lineage assignment, therapeutic-target assessment, and low-level disease reporting require different evidentiary thresholds.

## 2. Case presentation

### 2.1. Initial presentation and diagnosis

A 73-year-old woman presented on November 25, 2024, with fatigue, leukopenia, and anemia. Bone marrow contained 79.0% abnormal cells. Multiparameter flow cytometry (MFC) identified 66.4% B-lymphoblasts expressing cytoplasmic CD79a and terminal deoxynucleotidyl transferase, with partial surface CD19 and CD10 expression and weak surface CD22 expression. Surface immunoglobulin and light-chain restriction were absent. A minor t(8;14)(q24.2;q32) clone was identified by karyotyping, but fluorescence in situ hybridization did not confirm MYC rearrangement or MYC/IGH fusion. The integrated diagnosis was BCP-ALL.

### 2.2. Blinatumomab exposure and subsequent disease course

Blinatumomab was administered from December 6, 2024, at 9 µg/d on days 1–6 and 28 µg/d on days 7–14. The reviewed records did not document the rationale for the initial treatment selection or for the abbreviated course. Fever and transient speech and mental-status disturbances occurred on day 1, but the available record did not permit retrospective grading of cytokine-release syndrome or immune effector cell-associated neurotoxicity syndrome. On December 19, marrow blasts or immature lymphoid cells accounted for 2.5%, and MFC detected 0.02% B-lymphoblasts. On January 20, 2025, the recorded MFC value was 0.35%. She subsequently received weekly vincristine plus prednisone and then had a prolonged gap in specialist follow-up.

### 2.3. Relapse assessment and exclusion of MPAL

On January 29, 2026, marrow relapse was documented, with 60.0% blasts or immature lymphoid cells and 29.3% abnormal immature B-lineage cells by MFC. Relapsed cells retained cytoplasmic CD19 and CD79a, lacked surface CD19, and showed weak surface CD22 expression with CD13, CD33, partial CD15, and HLA-DR expression. They were negative for terminal deoxynucleotidyl transferase, CD10, cytoplasmic MPO, CD14, and CD64. The phenotype was interpreted as relapsed BCP-ALL with aberrant myeloid-associated antigen expression rather than MPAL. Clinical and immunophenotypic time points are summarized in Table 1, and representative clinical MFC plots are shown in Figure 1.

**Table 1:** Chronology, immunophenotype, and assay-qualified interpretation.

Time point	Clinical and flow-cytometry findings	Assay-qualified interpretation
November 25, 2024 Diagnosis	79.0% abnormal cells; 66.4% B-lymphoblasts by MFC. cCD79a+, terminal deoxynucleotidyl transferase+, partial surface CD19/CD10, weak surface CD22; no surface immunoglobulin or light-chain restriction.	BCP-ALL. Minor t(8;14) clone was not confirmed as MYC/IGH by FISH.
December 6–19, 2024 Blinatumomab	9 µg/d (days 1–6), then 28 µg/d (days 7–14). Day-1 fever and transient neurologic symptoms. The reason for treatment selection and abbreviated exposure was not documented in the reviewed records.	Formal cytokine-release syndrome/immune effector cell-associated neurotoxicity syndrome grading and treatment-selection rationale cannot be reconstructed.
January 29, 2026 Relapse	60.0% marrow blasts/immature lymphoid cells; 29.3% abnormal cells by MFC. cCD19+/cCD79a+, surface CD19-, weak surface CD22+, CD13+/CD33+, partial CD15+; cytoplasmic MPO-/CD14-/CD64-.	Relapsed BCP-ALL with myeloid-associated antigen expression; available data do not fulfill MPAL criteria.
February 6, 13, 20, 2026 INO salvage	Recorded as 1 mg each administration; body surface area unavailable. Relapse had weak surface CD22 expression. Complete liver-function, toxicity, and tolerability monitoring records were unavailable.	CD22-directed salvage rationale; mg/m^2^ dose and treatment tolerance cannot be reconstructed.
February 27, 2026 Post-INO MFC	0.5% marrow blasts; 0.007% low-level abnormal population (approximately 30 events) among 425,047 events, 7 days after the last recorded INO dose. Phenotype was descriptive: consistent with cCD19/cCD22/cCD79a/terminal deoxynucleotidyl transferase/CD13/CD33; surface CD22 was not detected with S-HCL-1.	0.007% is above the laboratory-reported LOD (0.0047%, approximately 20 events) and below the laboratory-reported LOQ (0.0118%, approximately 50 events): detectable but not precisely quantifiable. Complete gating and specimen-quality records were unavailable; no inference of CD22 loss was made.
April 21, 2026 Follow-up	No prior leukemia-associated population detected; surface CD19+/CD10+ hematogones 2.71%; 46,XX (20 metaphases).	Short-term morphologic and flow-cytometric improvement; not proof of durable measurable residual disease negativity or of treatment tolerability.
May 6, 2026 Last verified follow-up	Severe cytopenia on readmission; no new diagnostic MFC result.	Outcome duration and toxicity attribution remain uncertain.

The LOD is the lowest level distinguishable from analytical background; the LOQ is the lowest level measurable with prespecified precision.

BCP-ALL = B-cell precursor acute lymphoblastic leukemia, FISH = fluorescence in situ hybridization, INO = inotuzumab ozogamicin, LOD = limit of detection, LOQ = limit of quantification, MFC = multiparameter flow cytometry, MPAL = mixed-phenotype acute leukemia.

**Figure 1.**
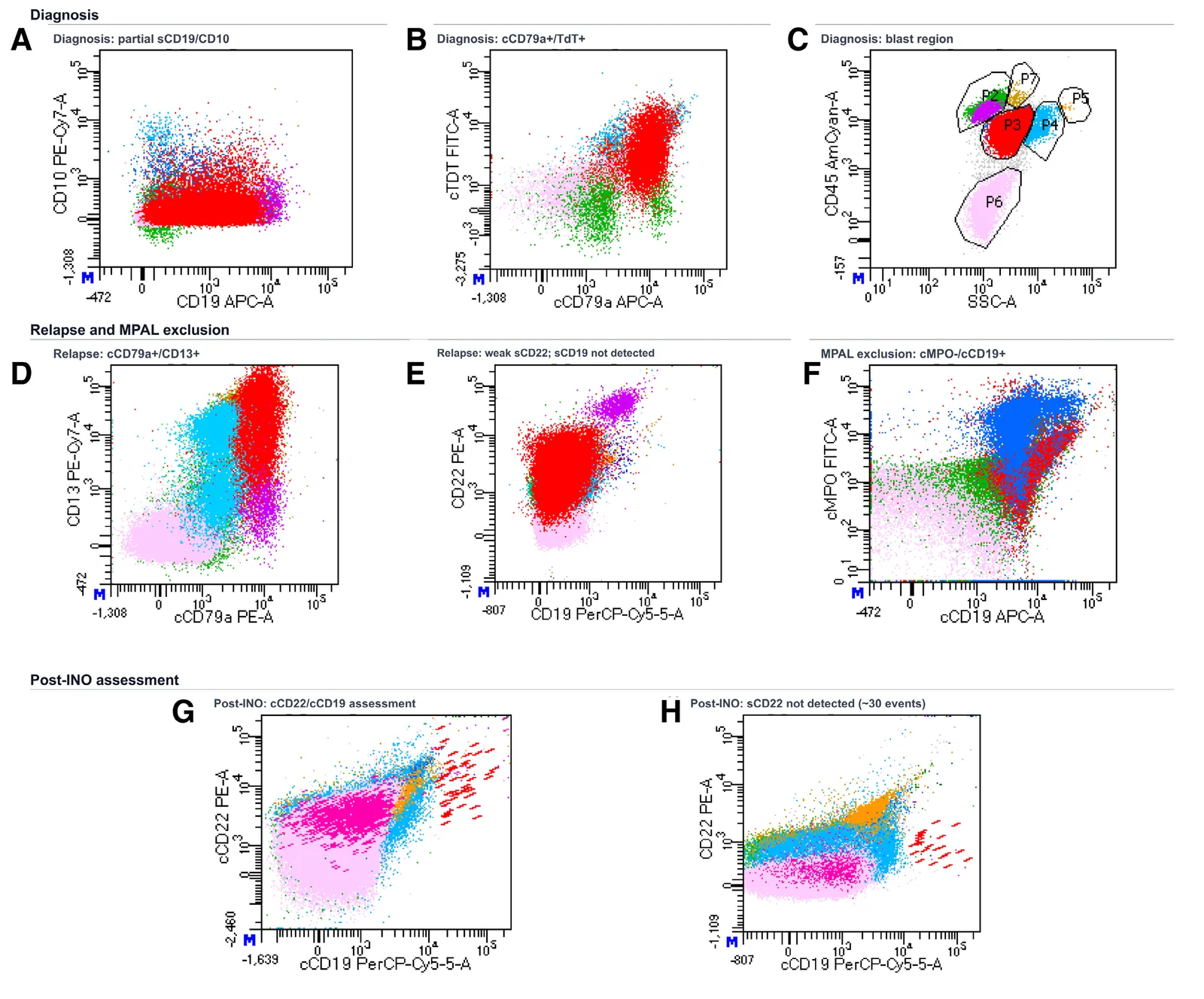
Representative clinical multiparameter flow-cytometry plots across diagnosis, relapse, MPAL exclusion, and post-INO assessment. Brief annotations within the figure identify each clinical and interpretive panel. (A) Diagnosis: partial surface CD19/CD10 expression. (B) Diagnosis: cytoplasmic CD79a and TdT co-positivity. (C) Diagnosis: CD45/side-scatter blast region. (D) Relapse: acquired CD13 expression with retained cytoplasmic CD79a. (E) Relapse: weak surface CD22 with surface CD19 not detected. (F) MPAL exclusion: cytoplasmic MPO was not detected while cytoplasmic CD19 was retained; the available findings did not support myeloid lineage assignment. (G) Post-INO assessment, 7 days after the last recorded INO dose: cytoplasmic CD22/cytoplasmic CD19. (H) Post-INO assessment: surface CD22 was not detected with the S-HCL-1 assay on the approximately 30-event abnormal population; this assay-specific finding does not establish CD22 antigen loss. Colored populations and gates reproduce the clinical analysis display; no new gates were created for this report. All patient identifiers were removed. c = cytoplasmic, INO = inotuzumab ozogamicin, MFC = multiparameter flow cytometry, MPAL = mixed-phenotype acute leukemia, MPO = myeloperoxidase, s = surface, TdT = terminal deoxynucleotidyl transferase.

### 2.4. INO salvage and posttreatment MFC

INO-based salvage was administered on February 6, 13, and 20, 2026, at 1 mg per administration. Body surface area and mg/m^2^ dosing could not be reconstructed. The reviewed records did not include complete treatment-tolerance, liver-function, or adverse-event monitoring data. MFC was performed on a BD FACSCanto II 8-color platform with BD FACSDiva 3.0 (Biosciences). Surface markers were assessed on unpermeabilized cells, whereas cytoplasmic markers were assessed after fixation and permeabilization. Surface CD22 was detected with clone S-HCL-1.

On February 27, 7 days after the last recorded INO dose, marrow blasts accounted for 0.5%. Among 425,047 acquired nucleated events, a low-level abnormal population of 0.007% (approximately 30 events) was identified. The laboratory-reported LOD was 0.0047% (approximately 20 events), and the LOQ was 0.0118% (approximately 50 events). The LOD represents the lowest level distinguishable from analytical background, whereas the LOQ represents the lowest level measurable with prespecified precision. Accordingly, the 0.007% population was detectable under the reported assay conditions but was not reliably quantifiable. No formal assessment of specimen quality or hemodilution, complete antibody and clone list, or full gating documentation was available. Its phenotype was therefore interpreted descriptively and was consistent with cytoplasmic CD19, cytoplasmic CD22, cytoplasmic CD79a, terminal deoxynucleotidyl transferase, CD13, and CD33 expression. Surface CD22 was not detected under the reported assay conditions. Whether recent INO exposure affected S-HCL-1 binding could not be assessed; therefore, no inference regarding CD22 antigen loss was made.

### 2.5. Follow-up

On April 21, 2026, no previously identified leukemia-associated abnormal population was detected under the reported assay conditions. Regenerating surface CD19-positive/CD10-positive hematogones accounted for 2.71%, and the karyotype was 46,XX in 20 metaphases. She was readmitted on May 6 with severe cytopenia, which marked the last verified follow-up. The available extract did not permit assessment of treatment adherence, complete tolerability, or the relationship between cytopenia and INO.

## 3. Discussion

The main contribution of this case is interpretive rather than mechanistic. Relapse after CD19-directed therapy should not be assessed solely against the diagnostic phenotype. In this patient, cytoplasmic CD19 and CD79a supported B-lineage differentiation despite the absence of surface CD19, whereas weak surface CD22 expression provided a clinical rationale for CD22-directed salvage. The temporal sequence does not establish that abbreviated blinatumomab exposure caused the altered phenotype. Subsequent vincristine/prednisone, a prolonged follow-up gap, and unmeasured biological evolution are competing explanations.^[5–7]^ A myeloid phenotype has been reported after CD19 CAR T-cell therapy in MLL-rearranged B-ALL, but that distinct therapeutic and biological context does not establish an analogous process in this patient.^[10]^

From a therapeutic perspective, cytoplasmic CD19 or CD22 positivity may support B-lineage assignment but does not by itself demonstrate the presence of an accessible surface target. Because blinatumomab and INO depend on surface CD19 and CD22, respectively,^[1,3]^ eligibility for retreatment or another antigen-directed therapy should not be inferred from cytoplasmic positivity alone. Such decisions should integrate contemporaneous surface-antigen testing, the antibody clone and epitope used, specimen quality, timing relative to therapy, and, when feasible, repeat or orthogonal testing.

The relapse phenotype also illustrates a diagnostic pitfall. CD13, CD33, and partial CD15 are myeloid-associated antigens, but MPAL requires MPO positivity or convincing monocytic differentiation for myeloid lineage assignment.^[11,12]^ The absence of cytoplasmic MPO, CD14, and CD64 did not support MPAL in this patient. Reporting CD13/CD33 positivity without the corresponding lineage-defining evidence would have overstated its diagnostic significance.

Similarly, the post-INO population of 0.007% is best described as a detectable low-level abnormal population below the laboratory-reported LOQ, rather than as a precise measurable residual disease endpoint. Because complete validation and gating records were unavailable, its phenotype remains qualitative. The later result indicates that no previously identified leukemia-associated population was detected under the reported assay conditions; it does not demonstrate durable measurable residual disease negativity.^[13,14]^

The absence of detectable surface CD22 on the post-INO population should not be equated with complete CD22 loss. Reduced surface CD22 expression and CD22-dim subpopulations have been reported after INO, and several genomic resistance mechanisms, including epitope-altering CD22 variants, have been described.^[8,9]^ However, neither those mechanisms nor an effect of recent INO on S-HCL-1 binding can be assigned to this patient without paired molecular testing or direct epitope verification. Treatment tolerance, including liver toxicity, and the contributions of comorbidity, infection, or limited marrow reserve to later cytopenias could not be fully assessed.^[3,15]^

## 4. Limitations

This single case cannot establish a clonal mechanism, antigen loss, or treatment causality. The reviewed records lacked a complete treatment-selection history, the reason for abbreviated blinatumomab exposure, body-surface-area data, paired molecular testing, a complete antibody and clone list, assessment of specimen quality or hemodilution, flow controls and full gating documentation, a complete INO toxicity-monitoring record, and longer outcome follow-up. The post-INO abnormal population was below the laboratory-reported LOQ. Its phenotype is therefore reported deliberately as qualitative and assay specific, rather than as definitive antigen loss, lineage switch, or a quantitative measurable residual disease endpoint.

## 5. Conclusion

Relapsed BCP-ALL after sequential CD19- and CD22-directed therapy requires repeated, assay-aware MFC interpretation. Surface and cytoplasmic CD19/CD22 should be reported separately, and CD13/CD33 expression alone should not be used to diagnose MPAL. In this patient, post-INO assessment showed morphologic improvement and a low-level abnormal population below the laboratory-reported LOQ. The surface CD22 finding, treatment tolerance, and duration of disease control remain uncertain.

## Author contributions

**Conceptualization:** Fangxiong Zheng, Zhongxin Lu.

**Data curation:** Fangxiong Zheng, Juan Dong, Xiaocui Cao, Shuangzhe Li, Tengfei Ma, Jiayi Li, Jinxi Wang, Yuchen Zhang, Zhongxin Lu.

**Formal analysis:** Juan Dong.

**Investigation:** Juan Dong.

**Validation:** Juan Dong.

**Writing – original draft:** Fangxiong Zheng, Juan Dong.

**Writing – review & editing:** Juan Dong.
